# New perspectives on vitamin D food fortification based on a modeling of 25(OH)D concentrations

**DOI:** 10.1186/1475-2891-12-151

**Published:** 2013-11-21

**Authors:** Jonathan Brown, Arne Sandmann, Anita Ignatius, Michael Amling, Florian Barvencik

**Affiliations:** 1 Department of Osteology and Biomechanics, University Medical Center Hamburg-Eppendorf, Martinistraße 52, D-20246 Hamburg, Germany; 2 Institute of Orthopaedic Research and Biomechanics, Ulm University, Helmholtzstraße 14, D-89081 Ulm, Germany

**Keywords:** Vitamin D, Vitamin D deficiency, Vitamin D food fortification

## Abstract

**Background:**

In Germany, vitamin D intake from food and synthesis in the skin is low, which leads to low 25(OH)D serum concentrations. In contrast to many other countries, general vitamin D food fortification is still prohibited in Germany, although the European Commission published a regulatory framework to harmonize addition of vitamins to foods. Thus the purpose of our study was to develop a vitamin D fortification model, taking into account all vitamin D sources with the goal to fulfill requirements of intake recommendations or preferable 25(OH)D serum concentrations. Finally, the aim was to assess the suitability of different carriers and associated risks.

**Methods:**

We developed a mathematical bottom-up model of 25(OH)D serum concentrations based on data about vitamin D sources of the German population such as sunlight, food and supplements for all federal states taking seasonal and geographical variations into account. We used this model to calculate the optimal fortification levels of different vitamin D carriers in two approaches. First we calculated required fortification levels based on fixed intake recommendations from e.g. the IOM or the DGE and second based on achieving certain 25(OH)D serum concentrations.

**Results:**

To lift 25(OH)D serum concentration in Germany to 75 nmol/L, e.g. 100 g bread has to be fortified with 11.3 μg during winter, resulting in a daily vitamin D intake of 23.7 μg. Bread seems to be a suitable carrier for base supply. However, overdose risk with a single fortified product is higher than the risk with several fortified carriers.

**Conclusions:**

With the model in hand, it is possible to conceive vitamin D fortification strategies for different foodstuffs and model its impact on 25(OH)D serum concentrations.

## Introduction

Sufficient vitamin D intake as well as adequate vitamin D synthesis in the skin is required to control calcium homeostasis a bone turnover. The effects of vitamin D on human health are diverse [1] but not yet fully investigated. However, vitamin D has been implicated in the risk of overall mortality [2], cancer [3-12], diabetes [13-15], musculoskeletal disorders [16], mental [17] and physical performance [18], hypertension [19], cardiovascular diseases [20], and autoimmune diseases [19,21]. Although, many benefits of vitamin D are ubiquitously known, recommended intake (RI) and more importantly upper limits (UL) have to be considered to prevent adverse effects such as vitamin D intoxication. Intoxication may occur at 25(OH)D concentrations above 500 nmol/L [22], while 75 nmol/L are considered as adequate [23,24].

In Germany, vitamin D intake from natural food sources [25,26] as well as vitamin D synthesis in the skin is low [23], which subsequently leads to low 25(OH)D serum concentrations. In Germany this was once reported in a population study of Hintzpeter and co-workers [26] and is now detailed with a novel mathematical bottom-up model of 25(OH)D concentrations [27]. Building on this knowledge, the aim of our study was to develop a novel vitamin D fortification model, taking into account all vitamin D sources, different carrier products suitable for fortification and various fortification scenarios to fulfill requirements of risk considerations as well as both intake recommendations and 25-hydroxyvitamin D concentrations.

Earlier fortification models have been published by Flynn and co-workers [28], Rasmussen and co-workers [29] or by Hirvonen and co-workers [30]. Models from Flynn and Rasmussen consider the safe upper limit for vitamin D fortification per energy unit. While in the Flynn model only vitamin D intake from natural food sources is considered as the basis for estimating the fortification levels, the Rasmussen model also takes into account the vitamin D intake from supplements. Whereas these two models give fixed values based on equations, the Hirvonen model developed the association between the risk of exceeding the UL and the fortification level. This is important for risk managers in order to decide on the acceptable risk. Our model, however aimed to combine advantages of previous models and add another, yet unconsidered, but significant aspect to vitamin D fortification modeling. It includes not only food intake from natural food sources and supplemental habits, but also vitamin D synthesis in the skin. In order to allow risk considerations, we defined various fortification scenarios for different dietary intake of natural food sources (5^th^ percentile, mean and 95^th^ percentile). Still, we have to mention that our study does not include estimates on an individual level, as it only considers average data.

## Methods

### Model is based on three core dimensions

A bottom-up model of 25(OH)D serum concentrations [27] as a function of sun exposure, food and supplements predicts and considers both vitamin D sources and vitamin D status of the average population in Germany. Output values are 25(OH)D serum concentrations of an average German individual for each month of the year and for each German federal state. For detailed description of the model and its results please see “New perspectives on vitamin D sources in Germany based on a novel mathematical bottom-up model of 25(OH)D serum concentrations” [27].

**Figure 1:**
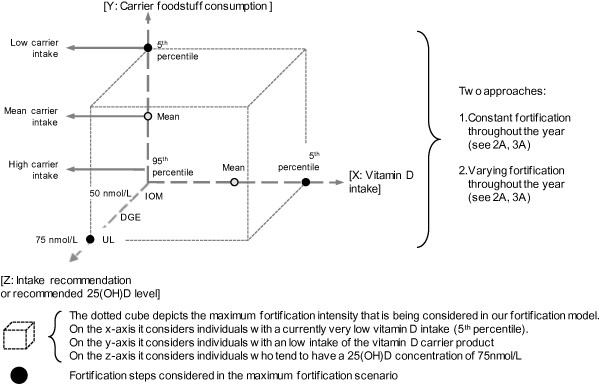
**Logic of the vitamin D fortification model.** The three axes depict the logic of each fortification intensity scenario considered in our model. The x-axis – vitamin D intake – represents daily vitamin D intake through natural food sources as well as supplements. While individuals of the 95^th^ (zero point) percentile already have a high vitamin D intake, individuals of the 5^th^ percentile (furthest from zero point) still have a low vitamin D intake. The more vitamin D an individual already consumes, the lower the calculated fortification intensity in our model.

Having a detailed understanding of the contributing factors and the resulting 25(OH)D concentrations, one can develop new perspectives on food fortification scenarios for Germany. The fortification model depicted in Figure 1 describes scenarios for a vitamin D fortification of bread, milk and juice. Zero point of each axis describes a minimum fortification scenario, while the fortification level rises with increasing distance from zero point. The maximum fortification scenario is indicated by the dotted cube. Depending on the chosen value of each axis, the size of the cube represents fortification intensity. This model does only stand for information for the average German population, but is not capable of considering fortification scenarios for individuals.

The x-axis – vitamin D intake – represents daily vitamin D intake through natural food sources as well as supplements [27]. While individuals of the 95^th^ (zero point) percentile have high vitamin D intake, individuals of the 5^th^ percentile (furthest from zero point) have low vitamin D intake. The more vitamin D the population consumes on average the less the food has to be fortified.

The y-axis – carrier foodstuff consumption – plots consumption habits of the general population for foodstuffs, which this model considers to be fortified. Zero point of y-axis belongs to the 95^th^ (high carrier intake scenario) percentile of food consumers who consume large quantities of considered fortified carriers and the 5^th^ (low carrier intake scenario) percentile, furthest from zero point, belongs to those who consume very little of respective foodstuff. The average consumption of considered foodstuff in the population is represented by the “mean carrier intake” scenario. As there are differences between men’s and women’s nutritional habits, in all “high carrier intake” and in all “mean carrier intake” case scenarios, higher consumption volumes and thus lower fortification levels were used for reasons of conservative considerations. In all regarded carriers, men are those who consume more than women. Only in the “low carrier intake” scenario, we used consumption quantities of those, who consume less. For all carriers considered this means women. An exemption to this is milk as in the 5^th^ percentile of milk consumers, men consume less than women. The “low carrier intake” (5^th^ percentile) scenario means that 95% of the individuals of the population would have an additional vitamin D intake through fortified food, which lifts their intake and thereby their 25(OH)D serum concentrations to a targeted level (z-axis).

The z-axis – intake recommendation or recommended 25(OH)D level – describes the recommended vitamin D intake or a 25(OH)D serum concentration to achieve. Zero point of z-axis refers to individuals, who tend to meet RI values of the Institute of Medicine (15 μg per day, IOM) and thereby reach 25(OH)D serum concentrations of 50 nmol/L which is defined as the lower value for adequate circulating 25(OH)D level by the IOM [31]. For the UL we considered people older than 8 years with an upper intake limit of 100 μg [31] or with a serum threshold of 75 nmol/L [23,24].

### Seasonally varying fortification introduced as alternative approach to constant fortification

This model is being developed in two approaches. The first approach aims to define a constant fortification (z-axis as intake recommendation) of different carrier foodstuffs throughout the year, as is common practice. For the constant fortification model, the vitamin D serum concentration model [27] was only used in parts. That is because 25(OH)D serum levels are not used as calculation base, but only vitamin D intake from natural food sources plus an average vitamin D intake from vitamin D supplements. To calculate constant fortification (f_c_) levels of carrier foodstuff, the difference (δ_i_) between recommended vitamin D intake (I_r_) and actual vitamin D intake through natural food sources and supplements (I_a_) is divided by intake of considered food to be fortified (F_i_), which are bread and milk as well as juice. This model can be easily adapted to all fortifiable food sources, but we only considered the three mentioned products. The underlying rationale of choosing these foodstuffs was on the one hand the goal to choose a carrier that is consumed by most people in Germany (here bread) and to choose carriers, for which there are many fortification experiences in other countries (here milk as well as juice).

The more the population consumes considered carrier foodstuff (F_i_), the less will it be fortified. The higher the recommended intake levels (I_r_), e.g. the 20 μg recommendation by the German Nutrition Society (DGE) [32], the more will the carrier be fortified. f_c_ is calculated for all scenarios, which are determined by the different characteristics of the three axes depicted in Figure 1. The dimension of f_c_ is μg per 100 g of considered fortified foodstuff, which is why we include the normalizing factor of 100 in the function f_c_. For bread, only bread and rolls were considered. Pastries such as baked goods, cakes, cream pies or pizza as well as cereal and grain products were not considered here. Among the category milk we subsumed milk, milk mix drinks as well as milk products such as yoghurt or buttermilk. Juices contain all fruit juices and fruit nectars, but not vegetable juices and juice drinks such as apple juice spritzer. For detailed input parameter of the fortification model, see Table 1[23,24,27,31-34].

$$f_{c}=\delta_{i}\cdot \frac{100}{F_{i}}=\left(I_{r}-I_{a}\right)\cdot \frac{100}{F_{i}}$$

**Table 1:** Input parameters of the model

**#**	**Parameter**	**Value or comment**	**Source**
1	**Vitamin D serum concentration model**		
1.1	25(OH)D concentration	Varies per month and per federal state of Germany [nmol/L]; average: 45 nmol/L	Brown et al [27].
1.2	Vitamin D intake through food	Varies per gender; mean average men: 3.4 μg and mean average women: 2.8 μg per day	Brown et al [27].
2	**Carrier foodstuff consumption**		
2.1	Bread	Men/Women	National Nutritional Survey II [34]
	5^th^ percentile	46 g/43 g	
	Mean intake	180 g/134 g	
	95^th^ percentile	377 g/270 g	
2.2	Milk	Men/Women	National Nutritional Survey II [34]
	5^th^ percentile	16 g/22 g	
	Mean intake	222 g/203 g	
	95^th^ percentile	712 g/555 g	
2.3	Juice	Men/Women	National Nutritional Survey II [34]
	5^th^ percentile	0 g/0 g	
	Mean intake	270 g/232 g	
	95^th^ percentile	1,200 g/1,000 g	
3	**Intake recommendation or 25(OH)D recommendation**		
3.1	Intake recommendation		
	IOM	15 μg	IOM [31]
	DGE	20 μg	DGE [32]
	Upper Limit (UL)	100 μg	IOM [31]
3.2.	Recommended 25(OH)D conc.		
	IOM	50 nmol/L	IOM [31]
	Bischoff-Ferrari et al.,	75 nmol/L	Bischoff-Ferrari et al [24],
	Domarus et al.		Domarus et al [23].
4	**Others**		
4.1	Conversion factor fortified food to 25(OH)D serum increase	2.32 nmol/L per 1 μg	O’Donnell [33]

The second approach aims to level 25(OH)D serum concentrations by varying the fortification amount per fortified foodstuff throughout the year (z-axis as 25(OH)D target level). For the varying fortification model, the vitamin D serum concentration model [27] was used as a whole. That is because 25(OH)D serum levels were used as calculation base. To calculate varying fortification levels f_v_ of the different carrier foodstuffs, the difference (δ_c_) between recommended 25(OH)D levels (L_r_) and actual 25(OH)D levels (L_a_) is divided by the conversion factor (c_f_) as well as by intake of considered food to be fortified (F_i_), which are bread and milk and juice. The conversion factor (cf) was derived from O’Donnell’s review on efficacy of food fortification on serum 25-hydroxyvitamin D concentrations [33]. O’Donnell included not only milk-based fortified food, but also different dairy based products, bread and orange juice, which reflects the vitamin D carrier portfolio chosen in our study. O’Donnell reported conversion factors indirectly as she mentions base and final 25(OH)D concentrations after a certain additional vitamin D intake. Out of 9 trials, 7 provided sufficient data for calculation of the absolute mean change from baseline in 25(OH)D. We used these 7 trials for conversion factor calculation for fortified food. The normalizing factor 100 is used likewise. Actual levels (L_a_) are specific for each month of the year and are derived from the vitamin D serum concentration model [27].

$$f_{v}=\delta_{c}\cdot \frac{100}{c_{f}\cdot F_{i}}=\left(L_{r}-L_{a}\right)\cdot \frac{100}{c_{f}\cdot F_{i}}$$

To get an impression of the new (“n”) 25(OH)D serum concentrations in case of vitamin D food fortification (L_nx_) for each month “x” of the year, we calculated resulting vitamin D concentrations as a function of actual (“a”) (25)OHD concentration per month “x” (L_ax_) plus its rise due to consumption of fortified food (F_i_). As a monthly change of the fortification intensity is not practical we defined cluster of seasonally changing fortification levels (summer time and winter time).

$$L_{\mathrm{nx}}=L_{\mathrm{ax}}+\frac{c_{f}\cdot F_{i}\cdot f_{v}}{100}$$

#### Software tools

All models were calculated using Excel version 2007. Macros were programmed in Visual Basic version 6.5. Pictures were created using PowerPoint version 2007 and Think-Cell version 5.2.

#### Ethics approval

This study was approved by the local ethics board of the University Medical Center Hamburg. There was no need for further ethics approval as the study is only based on publicly available data (see Table 1: Input parameters of the model). Hence there were no direct participants in our study which is why no written informed consent for participation in the study needed to be obtained.

## Results

### Ideal fortification levels vary by underlying conditions

**Figure 2:**
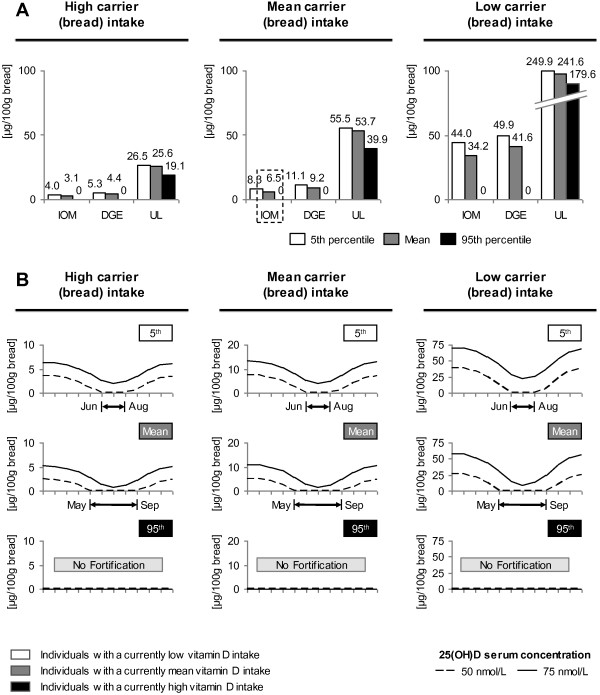
**Vitamin D fortification of bread.** Figure 2 depicts fortification levels for bread that are needed to either increase an individual’s vitamin D intake to a recommended level (IOM, DGE or to the UL, *figure****A***) or to increase an individual’s 25(OH)D concentration to a preferred level (50 nmol/L or 75 nmol/L, *figure****B***). The three columns represent the three scenarios of individuals with different carrier intakes (here: high, mean and low bread intake). **A.** Fortification levels for bread to meet intake values of nutritional guidelines of the IOM, of the DGE or to reach the UL. For definition of the “high carrier intake”, “mean carrier intake” and “low carrier intake” scenarios see Figure 1. Please note the break in the “low carrier intake” scenario for the UL. The x-axis depicts recommended intake levels, while y-axis shows fortification levels in μg per 100 g bread. White (5^th^ percentile), grey (mean intake) and black shaded (95^th^ percentile) bars reflect the current vitamin D intake levels (natural food sources and supplements). **B.** Fortification scenarios to reach concentrations of either 50 nmol/L or 75 nmol/L. The x-axis depicts varying fortification levels throughout the year, while the y-axis shows fortification levels in μg per 100 g bread. The dotted line belongs to individuals who tend to reach a 25(OH)D concentration of 50 nmol/L and the solid line belongs to the 75 nmol/L goal. Below each graph, months are indicated, during which no fortification is required. White (5^th^ percentile), grey (mean intake) and black (95^th^ percentile) labeled graphs stand for the current vitamin D intake levels (natural food sources and supplements).

**Figure 3:**
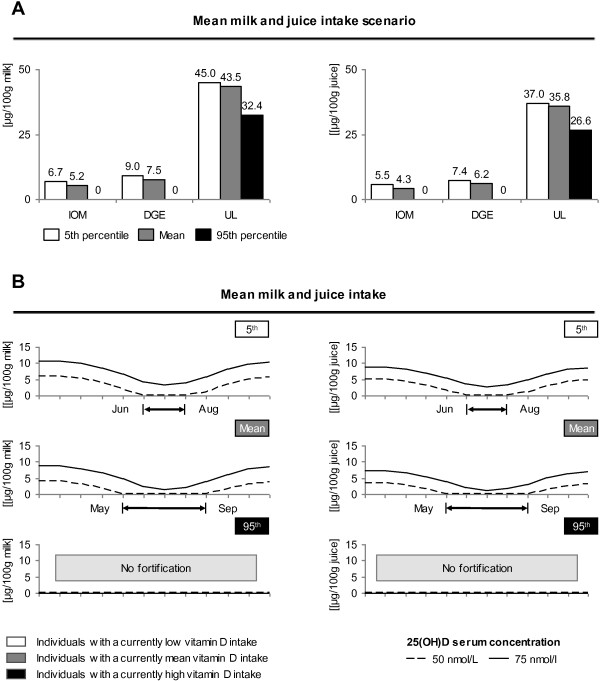
**Vitamin D fortification of milk and juice.** Figure 3 is analogous to Figure 2 and depicts fortification levels of milk and juice that are needed to either increase an individual’s vitamin D intake to a recommended level (IOM, DGE or to the UL, *figure****A***) or to increase an individual’s 25(OH)D concentration to a preferred level (50 nmol/L or 75 nmol/L, *figure****B***). The x-axis depicts recommended intake levels, while y-axis shows fortification levels in μg per 100 g of the respective carrier. White (5th percentile), grey (mean intake) and black shaded (95th percentile) bars reflect the current vitamin D intake levels (natural food sources and supplements). Shown here is only the scenario for individuals with a mean carrier (milk and juice) intake.

The Figure 2A/B portrays the vitamin D fortification level of 100 g of bread in the two approaches described in Figure 1. E.g. Figure 2A “mean carrier intake-IOM-mean scenario” (indicated as the dotted box in Figure 1 and Figure 2A) means that 100 g bread has to be constantly fortified with 6.5 μg in order to provide someone who is an average bread (“mean carrier intake”) and an average vitamin D consumer (“mean”) with 15 μg a day (“IOM”). The average bread intake is approximately 180 g, the average vitamin D intake is 3.1 μg (for both men and women). 6.5 μg multiplied with 1.8 (180 g divided by normalizing factor 100) plus 3.1 μg and 0.3 μg of supplements (men and women averaged) is approximately 15 μg (differences due to rounding). Figure 2B follows the same calculation logic, but is not based on a constant fortification level, but on seasonal variations of 25(OH)D concentrations. Scenarios of Figure 2B thus aim to keep vitamin serum concentrations on a constant level throughout the year. E.g. Figure 2B “mean carrier intake-75 nmol/L-mean scenario” means that in January, 100 g bread has to be fortified with approximately 11.3 μg. Someone who consumes 180 g bread (“mean carrier intake”) and who has an average vitamin D intake (“mean”) thus has a daily vitamin D intake need of approximately 23.7 μg (20.3 μg plus 3.1 μg plus 0.3 μg) to yield a 25(OH)D concentration of 75 nmol/L. We calculated fortification levels not only for bread (Figure 2A/B), but also for milk and juice, Figure 3A/B. In Figures 2B and 3B, we indicated months, during which no fortification is required in order to guarantee targeted 25(OH)D concentrations. In case fortification levels drop below the zero level, the fortification level was set to level 0. These months were the base for defining cluster of seasonally changing fortification levels.

### Effects on 25(OH)D concentration serve as basis for risk assessment

**Figure 4:**
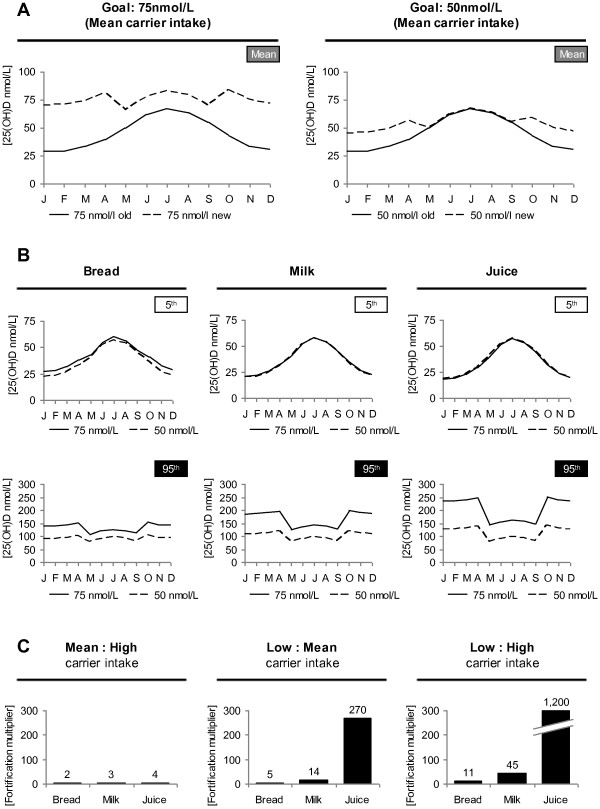
**Effect of food fortification on 25(OH)D concentrations and risk considerations. A.** Modeled effect of vitamin D food fortification on 25(OH)D concentrations. Portrayed is only the scenario of individuals with mean intake of vitamin D and the carrier product. The x-axis depicts varying fortification levels throughout the year, while the y-axis shows resulting 25(OH)D levels due to intake of vitamin D fortified food. The dotted line shows the effect of food fortification when aiming to level 25(OH)D concentrations at a certain value and the solid line shows 25(OH)D concentrations without food fortification. The left graph shows the 75 nmol/L goal, while the right graphs shows the 50 nmol/L goal. **B.** Effect of fortification on people with extreme dietary intake of vitamin D and the carrier product. Driving factors for these extreme estimates are high vitamin D intake (95th percentile) and high fortified foodstuff consumption (95th percentile) for the UL as well as low vitamin D intake (5th percentile) and low fortified foodstuff consumption (5th percentile) for the lower limit. Here, fortification levels are based on average carrier intake. The x- and y-axis are defined as in part A. The dotted line belongs to individuals who tend to reach a 25(OH)D concentration of 50 nmol/L and the solid line belongs to the 75 nmol/L goal. The three columns represent risk assessment for bread, milk, and juice. **C.** Comparison of effects of different vitamin D fortified carrier intake on vitamin D intake. Bars show, how much more vitamin D an individual from a certain carrier intake percentile would invest in comparison to another individual. Compared here are low (5th percentile) with mean and high (95th percentile) carrier intake. The x-axis shows different carrier products, while the y-axis represents multiplication factor of vitamin D intake due to vitamin D fortified food.

The modeled effect of vitamin D food fortification on 25(OH)D serum is shown in Figure 4A and Figure 4B. Figure 4A depicts the scenario of people who have an average vitamin D intake from food and supplements as well as an average consumption of the carrier foodstuff. The two graphs in Figure 4A are equal for all fortified foodstuffs, as our goal was to set 25(OH)D serum concentrations to the same level for each carrier product. In some months, the right graph shows no difference between old and new serum concentrations, as there is no fortification from May to September (see Figure 2B and 3B). For risk assessments, Figure 4B aims to assess the effect of a fortification level based on average carrier intake on people with extreme dietary intake of vitamin D as well as extreme dietary intake of the carrier product. Driving factors for these extreme estimates are high vitamin D intake (95^th^ percentile) and high fortified foodstuff consumption (95^th^ percentile) for the upper limit as well as low vitamin D intake (5^th^ percentile) and low fortified foodstuff consumption (5^th^ percentile) for the lower limit. It becomes obvious that bread has the most stable effect on 25(OH)D serum concentrations among the three foodstuffs when estimating to the upper or to the lower limit. Considering the upper limit, the effect of fortified food overrides all other vitamin D sources. This especially holds true for milk and juice. On the other side the lower limit considerations (5^th^ percentile) show almost no change, as this group of the population consumes almost none of respective foodstuffs. The effect of different consumption habits in the general German population for these three carriers is shown in Figure 4C. It shows vitamin D intake multipliers due to differences between 5^th^ percentile intake, mean intake and 95^th^ percentile intake. Very prominent is vitamin D intake multiplier of juice when comparing the “low carrier intake” with the “high carrier intake” scenario, which is due to the fact that the 5^th^ percentile of juice consumers is near 0 g per day. Most resistant against a change of the scenario is bread, which is due to the fact that the spread between low bread and high bread consumption is smaller than with any other considered carrier.

## Discussion

We compared the conventional approach of constant food fortification with a new one that takes into account seasonal variations of 25(OH)D concentrations. To our knowledge this is the first model that is able to find a fortification level, which is needed to either lift an average individual of the German population to a certain predefined 25(OH)D serum concentration or to raise the intake of an individual to a recommended intake. For means of risk assessments, this model considers several scenarios to estimate upper, mean and lower fortification levels for individuals with different intake. The novelty of the model in hand is based on the fact that it considers 25(OH)D serum concentrations rather than only intake recommendations and thus aims to level the 25(OH)D level throughout the year. Our model is programmed in a way that it can be easily adapted to all countries and all vitamin D carriers as long as input parameters are available for respective nations. Although the fortification model is based on simple mathematics, some aspects of the method of the model (4.1), the assumptions and input parameter (4.2) as well as the results (4.3) remain up for discussion and have to be further validated.

### The method of the model

Our approach is different to previously published models from Rasmussen et al. [29], Flynn et al. [28] or Hirvonen et al. [30] in two ways. First, these models add a specific level of vitamin D per 100 kcal. Our model adds a specific level of vitamin D per 100 g of food, but also considers intake of the German population. Due to the combination of intake in gram and fortification levels in vitamin D per 100 g, the calculation results in a similar logical approach, as our model is likewise able to define fortification levels for people with low, mean or high vitamin D intake. Second, our model not only considers fortification levels to meet certain intake levels, but also takes into account seasonal variations of 25(OH)D levels due to sun exposure. When making risk assessments, the second reason might be considered a shortcoming of our approach, as vitamin D synthesis in the skin may override the effects of nutritional and supplemental intake [35-37]. It could be thus raised to question, whether upper limit considerations are meaningful in the second approach. Opinions are divided concerning the contribution of sunlight as influencing factor for 25(OH)D serum concentrations. Diffey et al. [35] state that the sun may make up to 56% percent during summer times, while Shariari et al. [36] report that sun may contribute to vitamin D concentrations by more than 90%. As only average sun exposure habits are available as input parameter for Germany, risk assessment statements in the seasonal variations approach may be put up for debate.

### The assumptions and input parameter

Our model is based on a set of input parameter, see Table 1. While some parameters such as recommended intake levels are non-country specific, some parameters are, e.g. foodstuff consumption. When adapting our model to other countries the availability of these country specific parameters are a key prerequisite. Nevertheless, data availability might be a challenge in some countries.

We made every effort to refrain from input assumptions wherever possible. Nonetheless, an element of uncertainty remains the conversion factors of fortified food. In our model we used the systematic review of O’Donnell et al. [33] that determines the effects of vitamin D–fortified foods on serum 25-hydroxyvitamin D 25(OH)D concentrations, because the carrier products assessed in O’Donnell’s study match the vitamin D carrier portfolio chosen in our study. Yet these conversion factors have been subject of various discussions. Other researchers such as Vieth [38] claim that the conversion factor is lower at around 0.5-1.5 nmol/L per 1 μg vitamin D. However, our model is designed in a way that recalculation based on adapted input parameters, e.g. a lower conversion factor, can easily be performed.

### The results

The results of our fortification model (see example calculation in chapter “results”), stating that an average individual needs approximately 23.7 μg a day to reach a concentration of 75 nmol/L, are in line with other observations [7,11,24,39-44]. Other publications are of similar statements, proposing 25 μg to obtain an adequate serum 25(OH)D in the absence of UVB irradiance [45] or to raise the level by up to 25 nmol/L [46], which is comparable to our results. However the intake needed per day to reach a concentration of 75 nmol/L remains controversial since other publications suggest required intake levels of 40 μg per day and higher [47,48]. Furthermore it is important to note that determining fortification levels based on an average individual’s behavior implies that only a certain proportion of the entire population reaches the desired serum concentrations. If the goal is to lift the serum concentration of almost the entire population to a desired level, fortification levels have to be set much higher. However, in this case risk implications gain in importance.

Concerning food products to be fortified, one can argue, whether bread is a suitable carrier for vitamin D as there are only few, but promising experiences [49,50]. However from a nutritional point of view in Germany, bread makes sense in a couple of dimensions. Bread is a basic and a perishable foodstuff in Germany and it is the only food that does not show a consumption decline in the elderly population [34], which is of special importance to prevent osteoporosis. All age categories and all social classes consume bread and the difference between mean intake and the 95^th^ percentile is low compared to other potential vitamin D carriers [34]. This makes the amount of vitamin D intake through fortified foodstuff controllable. Additionally, bread is not a peak product such as juice (frequently consumed during some seasons, like summer time) that could potentially boost vitamin D concentrations to a maximum due to increased intake [34]. Hirvonen et al. [30] also show that bread is an efficient vitamin D carrier when looking for a solution to reduce the proportion of people with low vitamin D intake and which is safe in avoiding the risk of exceeding the UL. Still it remains open, whether vitamin D fortified bread alone can be the solution to alleviate vitamin D deficiency in Germany, as some studies show that food fortification with vitamin D is more efficient when a wide variety of foods are fortified with a low concentration [30,51]. The risk of overdose is higher for those, who consume larger quantities of certain foods, when only some foodstuff is fortified with high vitamin D concentrations [30]. The more food is fortified with lower concentration, the less likely is overdosing, as nobody can consume high quantities of all foodstuff that is fortified [30]. This is also in line with Välimäki and co-workers [51]. Considering the two other proposed foodstuffs to be fortified, milk as well as juices are common carriers for vitamin D [52-56], which however does not necessarily make these products suitable for fortification in Germany. A reason against milk and juice as carriers is the fact that the quantity spread of consumption for these foodstuffs is rather high [34]. In Finland, for example, this holds true for young women, who are not reached by the current milk fortification policy [57].

Bottom estimates (5^th^ percentile) in Figure 4B shows almost no difference for milk and juice, as the 5^th^ percentile almost consumes nothing of those carrier products. This does not hold true for bread as even the 5^th^ percentile consumes at least some bread. Top estimate (95^th^ percentile), reflects the quantity spread in consumption habits, especially for milk and juice. This is subsequently reflected in massive 25(OH)D concentration increase. One has to mention that these extreme estimates reflect very unlikely scenarios. However, these estimates are useful for risk considerations as they represent the maximum 25(OH)D concentration increase.

Regardless of the fortification strategy and its potential beneficial impact on the health of the general population, one has to keep in mind two things. First, food fortification per se is not allowed in Germany. There are only few exemptions allowed for general fortification. Among them are margarine, blended fat products as well as dietary food products. Second, vitamin D food fortification poses the risk of a vitamin D intoxication, though it appears to have been caused by excessive vitamin D fortification of dairy milk [58-60]. Furthermore, intoxication is not the only risk, which might go in hand with vitamin D food fortification. Although the therapeutic window for a safe supplementation of vitamin D is extremely wide, some groups could be at risk. The body regulates the biologic activation of cholecalciferol through control of 1α-hydroxylase activity [22]. This, however, does not apply for the safe supplementation of the active hormone (calcitriol) for example for people with chronic kidney disease, as the therapeutic window is relatively small here [61].

## Conclusions

We compared the conventional approach of constant food fortification with a new strategy that takes into account seasonal variations of 25(OH)D concentrations. We managed to show that bread as carrier product may be a suitable base. In terms of risk management, however, bread alone is probably not sufficient, as the risk of overdose with a single fortified product is higher than the risk with several fortified carriers [30,51]. Our model is programmed in a way that it can be easily adapted to all countries and all vitamin D carriers as long as input parameters are available for respective nations. To our knowledge, our model with its approach is unique and may help many countries, where the population is prone to vitamin D deficiency and which are searching for a strategy to improve the vitamin D status of their population to realize associated benefits [62]. General vitamin D intake and respectively 25(OH)D concentrations of the German population is low. A possible reason might be that food fortification is still prohibited in Germany. With this novel model in hand, it is possible to conceive vitamin D fortification strategies for different foodstuffs and model its impact on 25(OH)D concentrations. We propose to critically discuss the strategy of constant food fortification and show considerations for a seasonal variation of food fortification to balance 25(OH)D concentrations on an certain level.

## Abbreviations

DGE: German nutrition society (Deutsche Gesellschaft für Ernährung); IOM: Institute of Medicine; RI: Recommended intake; UL: Upper limits.

## Competing interests

The authors declare that they have no competing interests.
